# Research on Video Monitoring Technology for Galloping of OCS Additional Conductors of High-Speed Railway in Strong Wind Zone

**DOI:** 10.3390/s24237521

**Published:** 2024-11-25

**Authors:** Wentao Zhang, Wenhao Wang, Shanpeng Zhao, Huayu Yuan, Youpeng Zhang, Xiaotong Yao, Guangwu Chen

**Affiliations:** 1 School of Automatic & Electrical Engineering, Lanzhou Jiaotong University, Lanzhou 730070, China; zhangwentao@lzjtu.edu.cn (W.Z.);; 2 China Energy Engineering Group Gansu Electric Power Design Institute Co., Ltd., Lanzhou 730050, China

**Keywords:** strong wind zone, OCS, additional conductor, galloping monitoring, video image processing

## Abstract

The strong wind environment causes the additional conductor of the overhead contact system (OCS) of the Lanzhou–Xinjiang high-speed railway to gallop, significantly impacting the safe operation of the train. This paper presents the design of an online monitoring system for the galloping of additional conductors in the OCS, utilizing video monitoring for accurate and real-time assessment. Initially, the dynamics of the OCS additional conductor and its operational environment are examined, leading to the selection of suitable data transmission and power supply methods to finalize the camera configuration. Secondly, a preprocessing method for enhancing images of galloping in OCS additional conductors is developed, effectively reducing noise in edge detection through a region chain code clustering analysis. The video monitoring system effectively extracts wire edges, addressing the issues of splitting, breakage, and edge overlap in edge detection, while accurately identifying wire targets in video images. In conclusion, a galloping monitoring test platform is established to extract galloping data from additional conductors through video monitoring. The analysis of the galloping frequency and amplitude facilitates the comprehensive monitoring and assessment of the galloping status of OCS additional conductors. The video monitoring system effectively extracts and analyzes galloping data of the OCS additional conductor, fulfilling the fundamental requirements for the online monitoring of additional conductor galloping, and possesses significant engineering application value.

## 1. Introduction

**Figure 1:**
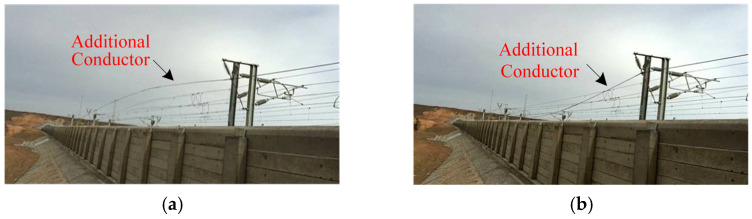
Additional conductor galloping of the Lanzhou–Xinjiang high-speed railway OCS in Baili wind zone. (**a**) Maximum galloping point; (**b**) Minimum galloping point.

**Figure 2:**
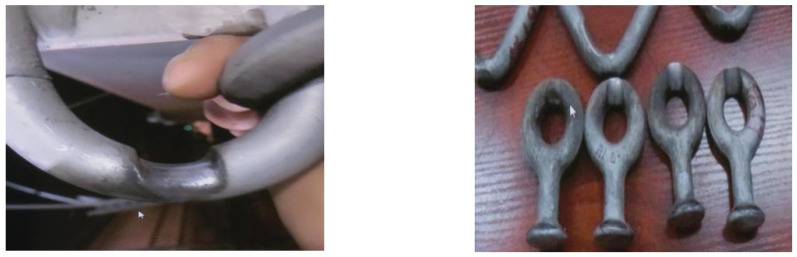
Fitting wear condition in strong wind zones.

The Lanzhou–Xinjiang high-speed railway extends 1775.779 km, traversing five significant wind zones in Gansu Province and the Xinjiang Autonomous Region of China, with these zones comprising 32.6% of the overall length. The greatest wind speed may reach 60 m/s, generating exceedingly potent immediate destructive forces. Windbreak barriers have been erected along the railway to prevent trains from being overturned by severe gusts, ensuring the safe running of 18 trains [1,2]. The additional OCS conductors of the Lanzhou–Xinjiang high-speed railway are affixed externally to the OCS support pillars without tension compensation and are situated in the wake acceleration zone behind the windbreak walls. Consequently, these supplementary conductors are particularly prone to conductor galloping [3]. Figure 1 illustrates the galloping of supplementary conductors in the Baili wind zone of the Lanzhou–Xinjiang high-speed railway overhead contact system (OCS). Figure 1 illustrates that, when the positive feeder attains its maximum and minimum galloping amplitudes, the contact wire exhibits no concurrent galloping. The substantial galloping of the additional conductors aggravated the abrasion of the conductor and fittings, leading to the occurrence of faults such as conductor breakage, dropping and discharge [4]. The deterioration of the fittings is illustrated in Figure 2. This creates multiple uncertainties regarding train operations, significantly upsetting the normal transit order and presenting considerable challenges to the safe and steady functioning of the railway.

The conventional method involves routine inspections of the OCS along railway lines predominantly through human patrols. Nonetheless, manual inspections are inefficient, labor-intensive, and significantly affected by the surrounding environment and weather conditions along the railway. An online video surveillance system has been developed to collect real-time data on the galloping of additional conductors in the OCS. This technology enhances the inspection efficiency and facilitates prompt reactions to existing conductor galloping occurrences.

In recent years, the swift advancement of intelligent video monitoring technologies, drones, and 5G base stations has led to the growing application of new technologies in the surveillance of transmission conductors. MAHAJAN et al. advocated for the installation of satellite positioning devices on transmission conductors to obtain real-time satellite navigation positioning data and carrier phase information. They integrated these data with current neural network technology to describe the trajectory of conductor galloping and acquired pertinent data [5]. Zhang Zihao and colleagues developed a transmission line galloping monitoring system utilizing the Beidou satellite navigation system, incorporating single-frequency dynamic post−processing (PPK) technology to ascertain the trajectory points of transmission line galloping [6]. REN H. P. et al. suggested an embedded picture monitoring system utilizing a digital signal processor (DSP) for the surveillance of transmission lines; nevertheless, it only offered a qualitative assessment of the conductors’ galloping state without delivering precise data [7]. Ren Zhidong, Ni Pan, and colleagues developed a galloping monitoring system utilizing airborne image processing, capitalizing on the contemporary application of drones for transmission line inspection. They devised image processing algorithms and edge feature recognition techniques to assess the conductor’s galloping state in video images and extract pertinent data [8,9]. Li Z. et al. devised a high−precision target recognition method utilizing an enhanced YOLO v10 model and established an autonomous inspection system for high-voltage transmission lines employing drones, markedly improving inspection efficiency [10]. Yan Li et al. proposed a method utilizing artificial intelligence to detect conductor galloping in transmission lines by deploying accelerometers, fiber optic sensors, and cameras to gather real-time data on line vibrations, displacements, and other parameters, subsequently analyzing the conductor galloping state through machine learning algorithms [11]. Elhanashi et al. introduced an edge AI algorithm that integrates thermal imaging cameras, visible light cameras, and YOLOv4-tiny detectors for real-time distance monitoring. The methodology of developing efficient algorithms for limited contexts offers valuable insights for our real-time monitoring system for additional conductor galloping [12]. N. Ye et al. introduced a method termed OoD-Control to attain control generalization in unfamiliar contexts. This method theoretically guarantees the performance of control algorithms across diverse environments by introducing random noise and practically confirms its efficacy, providing a new viewpoint for improving the generalization ability of control algorithms [13].

In summary, the installation of accelerometers on conductors to measure displacement, frequency, and other characteristics associated with conductor galloping constitutes a contact−based monitoring technique. Nevertheless, these sensors may influence the initial galloping condition of the conductors, resulting in a certain degree of divergence in the monitoring data. Drone-based inspections of transmission lines generally seek to assess the state of the lines and related equipment, providing substantial efficiency enhancements compared to manual inspections; yet, certain disadvantages persist. The mobility of drones complicates the accurate monitoring of conductor galloping. Drones currently lack the capability for real-time deep processing of video pictures, hence constraining their ability to accurately represent the actual galloping status of conductors. Environmental limitations exist: adverse environmental circumstances, such as high winds or ice and snow on railway lines, might detrimentally affect drone operations and, subsequently, the dependability of the monitoring procedure. Due to the rising demands for the intelligent operation and maintenance of railway power supply systems, the necessity for efficient, precise, and real-time monitoring of conductor galloping has become increasingly critical.

This article proposes a video monitoring system for the galloping of OCS extra conductors, identifying suitable data transmission and power supply techniques, and finalizing the camera installation. The system encompasses the creation of a picture preprocessing and enhancement technique specifically designed for the insertion of new conductors, succeeded by edge detection and extraction of the conductors from the enhanced images. A laboratory galloping monitoring platform is established to assess the galloping state of the OCS extra conductors. The deployment of this online monitoring technology is crucial for guaranteeing the safe functioning of the railway and improving the sophistication of management inside the railway system.

## 2. Video Monitoring System Design: Analysis of On-Site Environment and Monitoring Objects

The Lanzhou–Xinjiang high-speed railway crosses three provinces: Xinjiang, Qinghai, and Gansu, and navigates five significant wind zones with intricate geographical conditions. The elements of the monitoring system must comply with the current regulations of the railway operations department, address the immediate requirements of train operations, and take into account the local natural environment and railway infrastructure. Furthermore, the system must guarantee that the galloping monitoring outcomes are precise, rapid, stable, and dependable.

### 2.1. Structure Analysis of the Lanzhou–Xinjiang High-Speed Railway OCS

**Figure 3:**
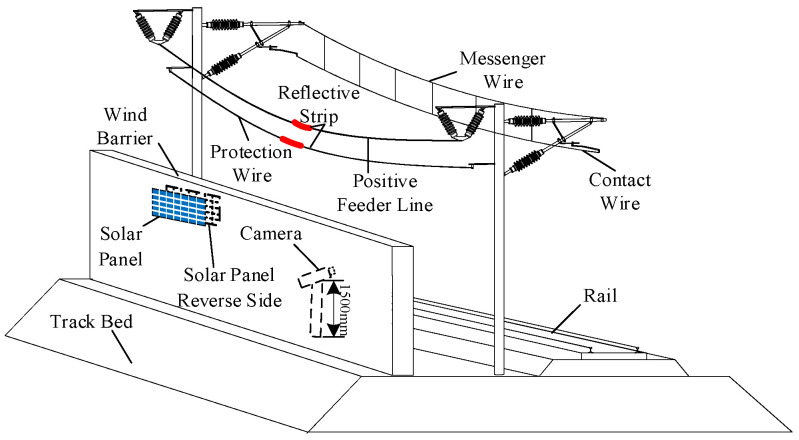
Structure diagram of the OCS and track system for the Lanzhou–Xinjiang high-speed railway in the strong wind zone.

Windbreak walls were engineered along the windward side of the Lanzhou–Xinjiang high-speed railway in areas prone to strong winds to mitigate the effects of severe winds, sand, and other natural calamities on trains and tracks. This design also seeks to avert train overturning, as illustrated in Figure 3. On-site investigations revealed three primary types of windbreak structures: embankment windbreak walls, windproof tunnels, and bridge windbreak screens, which collectively constitute 65% of the total length of the wind zone along the Lanzhou–Xinjiang high-speed railway.

Using the most prevalent embankment windbreak walls as a reference, these walls measure between 3.5 m and 4.0 m in height and are positioned on the windward side of the OCS support poles, which have an embankment height of 2.0 m and a support pole height of 12.0 m. The distance between two support poles varies from 40 m to 55 m, with the supplementary conductors situated at a height of 7.2 m above the embankment. The OCS supplementary conductors of the Lanzhou–Xinjiang high-speed railway have a unique structure and distribution pattern. This study’s monitoring system aims to minimize economic costs, alleviate data processing demands, and enhance operational efficiency by strategically positioning cameras in regions characterized by significant wind speed variations and pronounced conductor galloping, informed by the wind speed and topographical attributes of high-wind areas. The galloping status of conductors in these significant wind zones illustrates the general galloping behavior of conductors in the adjacent wind zones. Moreover, reflective strips are affixed at the midpoint of the monitored supplementary conductors to facilitate the identification of the target conductor amidst many conductors.

### 2.2. Analysis of the Galloping of Additional Conductors

**Figure 4:**
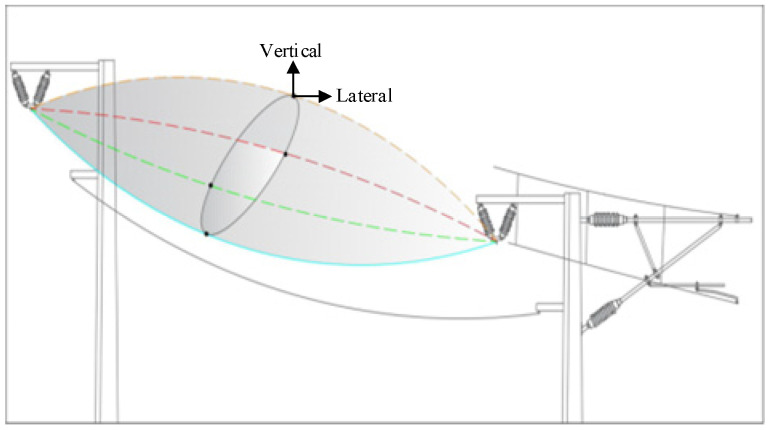
Displacement diagram of the galloping of OCS additional conductor.

In an electrified railway overhead contact system, the contact wire immediately connects with the pantograph of an electric locomotive, facilitating the passage of electrical energy. Moreover, auxiliary conductors, including supplementary conductors and protection wires, are essential for enhancing the electromagnetic field environment of the OCS, augmenting power supply capacity, and ensuring the safety of power delivery. The supplementary conductor is positioned on the field side of the support pillar [14]. Its uncomplicated structure and absence of tension compensation render it more susceptible to galloping. Figure 4 illustrates the rapid emergence of the OCS additional conductors on the Lanzhou–Xinjiang high-speed railway. The galloping of supplementary conductors results in heightened wear and tear on the line fittings along the route, as well as conductor strand fractures, which considerably disrupt the normal functioning of the railway. The galloping of supplementary conductors is generally a form of self-excited vibration [15]. The galloping phenomena develops progressively due to the wake flow generated by windbreak barriers. At reduced wind velocities, the conductors oscillate with minimal amplitude and low frequency around their equilibrium position. As wind speed escalates, the trajectory of movement alters—the horizontal displacement diminishes, frequency amplifies, and the amplitude of vertical movement progressively increases. Ultimately, the galloping stabilizes, resulting in an oval trajectory [16].

### 2.3. Galloping Monitoring Principle

A non−contact monitoring method was selected to gather galloping data, adhering to the idea of not influencing the movement of additional conductors in the OCS. Video image monitoring was used to enhance real-time surveillance in the intricate railway environment. The imaging model of the video surveillance terminal for physical objects adheres to a pinhole imaging model. To determine the characteristic points in the image, it is essential to establish the linkages among the world coordinate system, camera coordinate system, image coordinate system, and pixel coordinate system.

**Figure 5:**
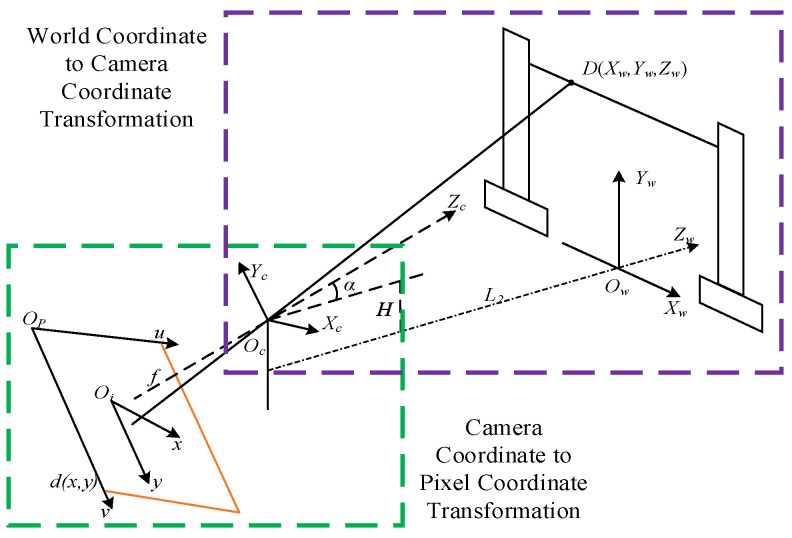
Schematic diagram of imaging of a point on the OCS additional conductor.

**Figure 6:**
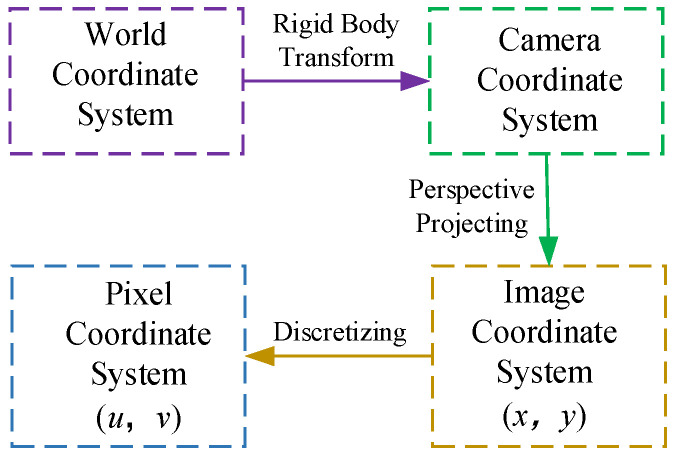
Coordinate transformation structure diagram.

The target measurement point coordinates $D(X_{w},Y_{w},Z_{w})$ on the OCS additional conductors are transformed through the coordinate systems to obtain their corresponding coordinates in the two-dimensional digital image, denoted as d(x,y). As illustrated in Figure 5, determining the coordinates of any point in space requires transformation through these coordinate systems, as shown in Figure 6.

By comprehending the correlation between the coordinates in the video image, these coordinates are manipulated to ascertain the locations of the supplementary conductors in the surveillance video. The displacement, frequency, and other metrics are examined and evaluated during the galloping of the additional conductors to finalize the monitoring of the conductors.

## 3. Design of a Video Monitoring System for Galloping Detection

A comprehensive investigation of the structure and environment of the supplementary conductors in the electrified railway OCS has led to the construction of a dependable, stable, safe, and cost-effective monitoring system for detecting conductor galloping, incorporating the following considerations:

(1) The system design must strive to minimize or eliminate modifications to the railway structure, guaranteeing no disruption to train operations.
(2) The monitoring system must guarantee that the installed equipment is stable, reliable, and capable of functioning properly even in adverse environmental conditions.
(3) Economic viability: the design of the monitoring system must account for economic considerations and correspond with the operational requirements of contemporary railway systems.
(4) Compliance with railway standards: the galloping monitoring system design should comply with railway design standards.

### 3.1. Overall Design of the Video Monitoring System

**Figure 7:**
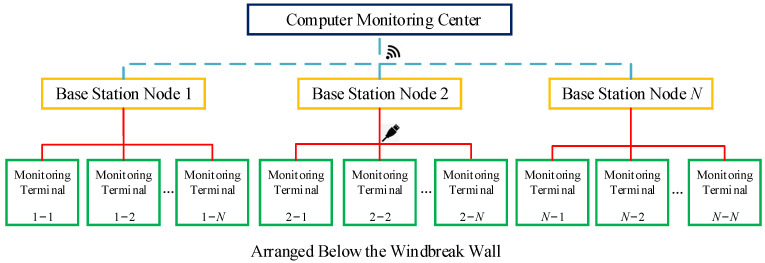
Design block diagram of additional conductor galloping monitoring system.

To efficiently obtain monitoring data, a monitoring video terminal is positioned alongside the supplementary conductors within each OCS span. These video terminals convey data across a wired network to a base station. The base station regulates a suitable quantity of monitoring terminals based on the surrounding environment. Each base station conveys monitoring data to the control center using a wireless network [17]. This system design guarantees the dependable and effective conveyance of data. Figure 7 depicts the detailed system design plan.

### 3.2. Selection of Data Transmission and Power Supply Methods

Data transfer from the base station to the monitoring center utilizes GPRS wireless technology, offering two primary options: public and private networks. Public network transmission is economical, utilizing the existing infrastructure of telecom operators, such as base stations and signal towers. Nevertheless, public network signals are predominantly focused in particular regions and do not include the entire railway network. Due to the majority of the Lanzhou–Xinjiang Railway being situated in the Gobi Desert’s high-wind zone, where signal coverage is inconsistent, a specialized private network has been implemented to ensure real-time, stable, and reliable data transfer [18]. The design may fully utilize the placement of the windbreak wall alongside the Lanzhou–Xinjiang high-speed railway OCS. Positioning the cameras on the leeward side of the wind barriers minimizes the influence of heavy winds on the cameras. The camera data are transferred to the monitoring center using a wireless network. Due to the severe wind and sand conditions along the railway, it is imperative to guarantee the safety and dependability of the transmission system. The wind barriers safeguard the trains and serve as a natural shield for the data transmission wires, improving their stability and protection against environmental elements.

Due to the minimal power demands of the video terminals, batteries are selected to energize the cameras. Table 1 delineates a survey and assessments of prevalent batteries now available on the market. In comparison to alternative battery types, lithium-ion batteries present distinct advantages regarding dimensions, thermal adaptability, and mass. Lithium-ion battery technology is well established, with a high energy density, substantial capacity, compact size, numerous charge–discharge cycles, and minimal environmental impact. To eliminate the necessity for the frequent manual replacement of individual lithium-ion batteries, a hybrid system comprising lithium-ion batteries and solar photovoltaic panels is selected to energize the video terminals. Simultaneously, the design of the monitoring terminal ensures that the video monitoring system activates real-time detection solely during conductor galloping events, hence minimizing power consumption and system degradation.

### 3.3. Video Monitoring Camera Arrangement

For precise target identification at extended ranges and in intricate situations, the camera must possess a resolution of no less than 8 megapixels (8MP). Furthermore, to accurately capture the dynamic movements of the conductors, the camera’s frame rate must be a minimum of 30 fps, guaranteeing the retention of essential frames throughout recording. The camera must possess superior low-light capabilities to obtain clear images in diverse lighting conditions, including overcast weather or nighttime, ensuring the effective functioning of the monitoring system.

The positioning of the camera is dictated by the geography, wind velocity data, and previous records of conductor galloping in high-wind areas. The system prioritizes monitoring portions susceptible to conductor galloping, particularly those experiencing the most severe instances. Each camera functions autonomously, recording real-time video data and relaying it to the backend system. A distributed design is utilized to mitigate the effects of single-point failures on the system. GPS-based time synchronization is employed across the system to guarantee the synchronized analysis of conductor galloping data, ensuring that video footage from various locations is aligned for coherent evaluation.

Upon the transmission of the data to the backend system, computer vision and image processing algorithms conduct real-time analysis of the video feed. These algorithms identify characteristics such as the amplitude and frequency of conductor galloping. Should the amplitude surpass a predetermined level, the system autonomously activates an alert to inform maintenance workers. All cameras are administered via a centralized control platform that facilitates remote modifications, system status monitoring, and troubleshooting. In the event of a malfunction, the system autonomously identifies the problem and generates an alarm, necessitating maintenance. The technology dynamically modifies the camera’s operational frequency based on real-time weather data, including wind speed and direction. During high-wind-speed intervals, the system can augment the video capture rate to obtain additional galloping data, whereas in lower-wind-speed conditions, it diminishes the capture rate to minimize superfluous data processing.

During camera installation, optimal shooting positions and focal lengths must be determined to guarantee that the cameras accurately focus on the target conductor. Appropriate camera angles reduce interference from additional conductors inside the monitoring perspective. A rectangular border can be established within the camera’s field of view using coordinates, and image processing will exclusively concentrate on this specified area, disregarding the remainder. Moreover, reflecting indicators like micro−LEDs are positioned at the midway of the designated conductor, utilizing optical qualities to differentiate the target conductor from others. The midpoint, being the locus of highest vibration amplitude, displays the most significant motion attributes. At this stage, monitoring data can reflect the conductor’s overall galloping behavior, yielding reliable and continuous information. This method guarantees that the camera accurately recognizes and focuses on the target conductor, even in intricate illumination or conductor overlap scenarios, assuring precision recognition.

**Figure 8:**
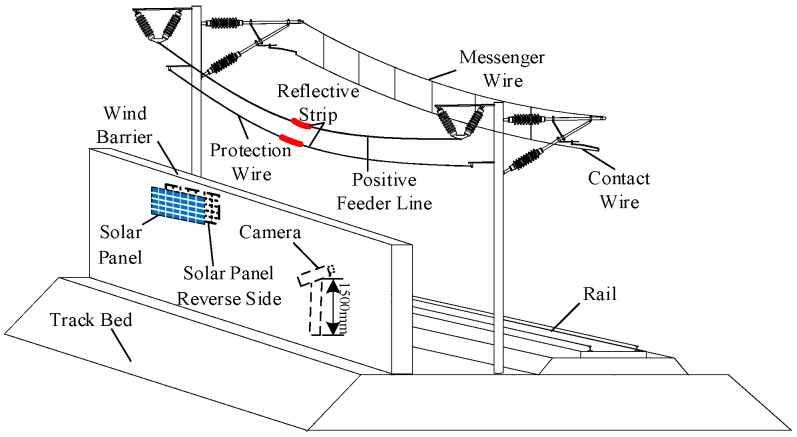
Schematic diagram of the terminal layout of the additional conductor galloping monitoring system.

Figure 8 depicts the configuration of the monitoring terminal for the conductor galloping of the OCS. The camera is positioned on the leeward side of the windbreak wall to see the galloping of the supplementary conductors, utilizing the wall as a shield against wind and sand. By modifying the camera’s elevation angle, the galloping condition of the supplementary conductors is recorded inside the video monitoring terminal’s field of view, facilitating the real-time surveillance of the OCS supplementary conductors on the Lanzhou–Xinjiang high-speed railway. Solar photovoltaic panels are affixed to the wind barriers, guaranteeing sufficient solar exposure without compromising the structural integrity of the railway on-site.

## 4. Image Processing and Edge Detection

**Figure 9:**
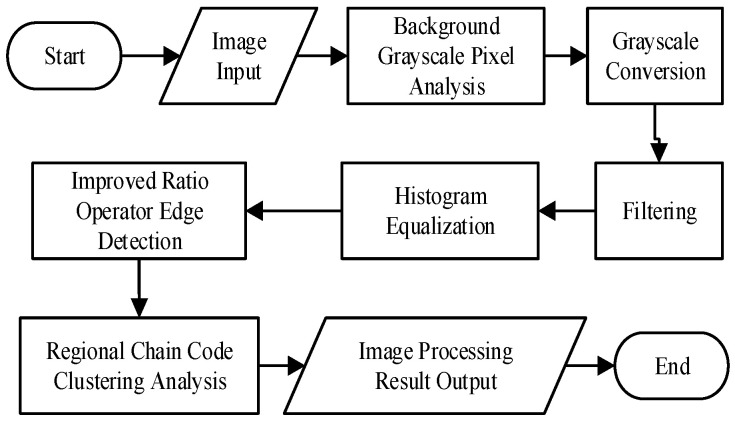
Image preprocessing and edge detection flowchart.

The camera surveillance of the conductor’s galloping on the OCS generates a substantial volume of sent data. To satisfy the real-time demands and hardware specifications while efficiently monitoring conductor galloping, it is essential to extract the conductors from the video images. Variations in the environment, weather, and shooting equipment, particularly under severe wind and sand conditions along the Lanzhou–Xinjiang high-speed railway, sometimes result in poor-quality photographs, complicating feature extraction. The pixel width of the supplementary conductors is minimal, and they constitute a negligible fraction of the image. Moreover, noise and several conditions during transmission exacerbate the extraction process. Consequently, digital images relayed by the monitoring terminal require preprocessing and enhancement. Subsequent to the picture preprocessing procedures, the targets of the supplementary conductors in the digital image are enhanced. The subsequent phase is identifying and extracting the edge features of the supplementary conductors in the image. Figure 9 depicts the workflow for picture preprocessing and edge detection.

### 4.1. Image Preprocessing Enhancement

**Figure 10:**
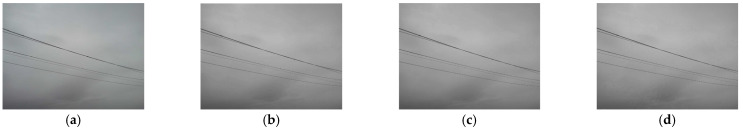
Image grayscale effect processing diagram. (**a**) Original image; (**b**) Average method; (**c**) Weighted average; (**d**) Maximum value method.

The images of the supplementary conductors relayed by the video monitoring terminal are in color, specifically utilizing the RGB color model. These images have substantial data volumes, consuming considerable connection capacity and resulting in sluggish transmission speeds and diminished efficiency. In galloping monitoring, color features are not critical for the analysis of the amplitude and frequency. Various grayscale levels can denote the chroma and brightness levels of the supplementary conductors in the image. Grayscale photos diminish the data volume and enhance the algorithm efficiency. A local backdrop image of the supplementary conductors is chosen and processed utilizing three grayscale techniques to transform the RGB color image into a grayscale image, as seen in Figure 10.

The application of the mean approach in image processing yields a smoother and more stable resultant image. The weighted mean processing elucidates greater edge features of the supplementary conductors upon meticulous examination. Maximum value processing generates a grayscale image characterized by enhanced color depth and increased brightness [19]. Given the attributes of the supplementary conductors and the ensuing preprocessing stages, the weighted mean method is selected as the most effective grayscale processing strategy.

**Figure 11:**
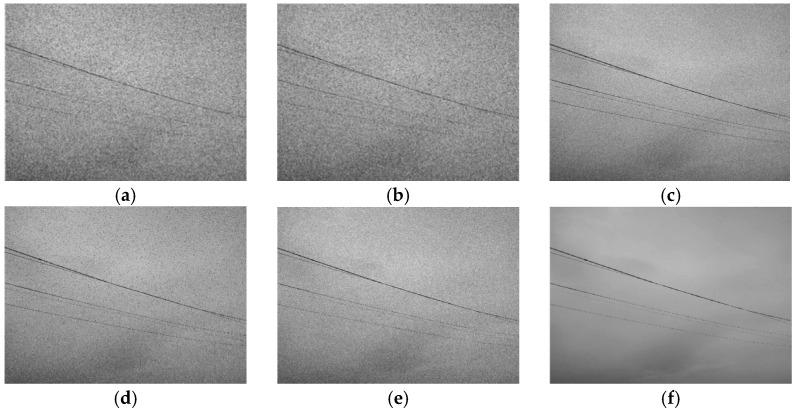
Different filtering methods. (**a**) Mean filtering—Gaussian noise processing. (**b**) Mean filtering—salt-and-pepper noise processing. (**c**) Gaussian filtering—Gaussian noise processing. (**d**) Gaussian filtering—salt-and-pepper noise processing. (**e**) Median filtering—Gaussian noise processing. (**f**) Median filtering—salt-and-pepper noise processing.

The images sent by the monitoring terminal are affected by the shooting environment and camera apparatus, potentially introducing various forms of noise during data transfer. This noise diminishes the quality of digital photographs, obscures the edge features of the conductors, and may result in problems such as edge overlap, multiple edges, and missed detections during the edge feature detection process. To prevent problems such as conductor overlap, duplicate edges, and missed detections in edge feature detection, while enhancing performance, filtering is implemented on the grayscale-processed pictures. Following the conversion of the images to grayscale, Gaussian noise and salt-and-pepper noise are introduced, after which, three widely utilized filtering techniques are applied to the grayscale images, as seen in Figure 11.

Post-filtering, noise induced by variables such as equipment temperature can be effectively eradicated from the image. However, during the image capture process, fluctuations in lighting may cause certain regions of the digital image to look dim. The video pictures relayed by the monitoring terminal exhibit environmental effects that render the grayscale values of the additional conductors and the backdrop nearly indistinguishable, complicating the precise detection and extraction of conductor targets by traditional image processing techniques. Consequently, image equalization is necessary.

**Figure 12:**
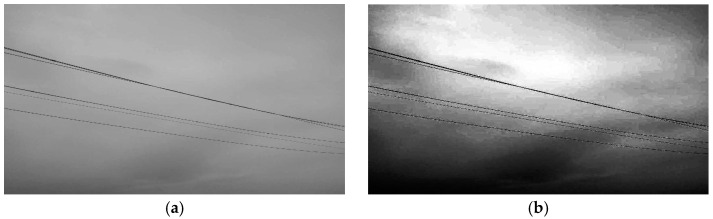

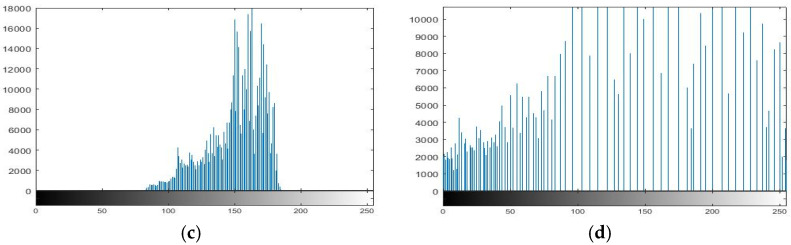
Image grayscale effect processing diagram. (**a**) Original image; (**b**) Equalized image; (**c**) Original image histogram; (**d**) Equalized image histogram.

The objective of picture equalization is to execute the nonlinear stretching of the entire image structure, reallocating the pixels to improve the contrast of the digital image [20]. Following grayscale conversion and filtering, the supplementary conductor targets in the image become more pronounced and distinct, facilitating the subsequent identification of edge characteristics. The efficacy of this technique is demonstrated in Figure 12.

### 4.2. Image Edge Feature Detection and Extraction

In complex backgrounds, the Ratio operator, a statistical model-based line edge detection algorithm, is highly effective in noise resistance and can accurately extract edge details of targets [21]. Due to the presence of background noise, some conductors in the image may appear blurred, overlapping, or broken, resulting in discontinuous segments after edge detection. The Ratio operator is applied to the preprocessed and enhanced target image to detect the grayscale range of additional conductors in complex backgrounds, reducing missed detections, false detections, and noise interference. A variable line feature detection threshold $l_{th}$ is introduced, and region chain code clustering is used to analyze the results of edge detection, ensuring the edges are accurate and complete.

**Figure 13:**
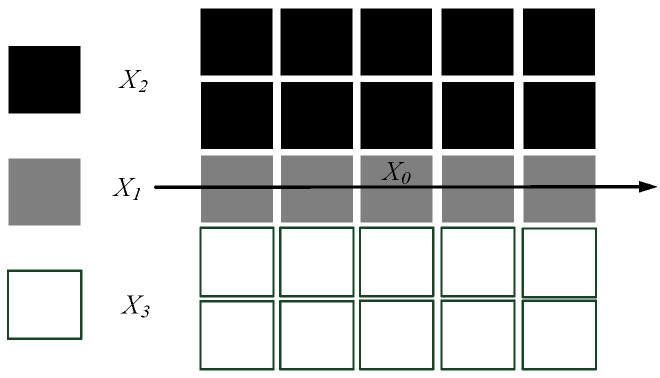
Horizontal Ratio operator.

The horizontal setting of the Ratio edge detection operator is illustrated in Figure 13. The target additional conductor $X_{0}$ is treated as the center point, and a 5 × 5 area around it is divided into three different rectangular windows. The gray boundary $X_{1}$ is a 1 × 5 rectangular window, while the black boundary $X_{2}$ and white boundary $X_{3}$ are 2 × 5 rectangular windows. By calculating the grayscale average of all the pixel values within $X_{1}$, $X_{2}$, and $X_{3}$, and comparing the grayscale average of the $X_{1}$ region with those of $X_{2}$ and $X_{3}$, it is determined whether the center target $X_{0}$ is a point on the linear structure of the conductor.

**Figure 14:**
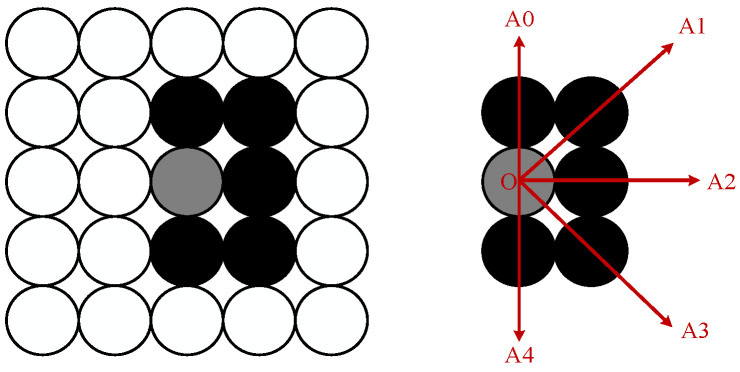
Four−way connectivity definition.

From the conductor image, it can be observed that most of the background noise is distributed on both sides of the conductor, with a few noise points scattered in other regions. The noise is often spread out with large gaps, covering a wide area and appearing relatively continuous. Considering the distribution characteristics of the conductor in the image, this paper employs a four-directional connected region clustering analysis method to classify the conductor edges and background noise. The four-directional chain code is defined as shown in Figure 14. The four−directional chain code considers a pixel point O as the center. If a pixel point A is located in any of its A1, A2, A3, or A4 positions, pixel point A is considered to belong to the same region as O.

**Figure 15:**
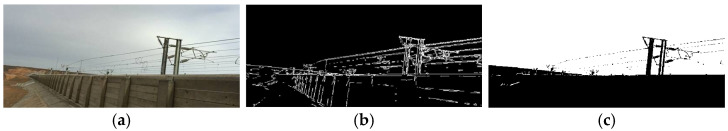
The experimental results of Scenario 1. (**a**) Original image. (**b**) Improved Ratio operator processing. (**c**) Four-direction chain code tracking processing.

**Figure 16:**
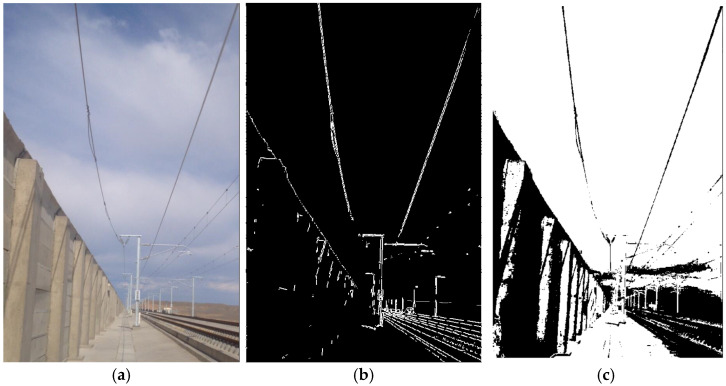
The experimental results of Scenario 2. (**a**) Original image. (**b**) Improved Ratio operator processing. (**c**) Four-direction chain code tracking processing.

**Figure 17:**
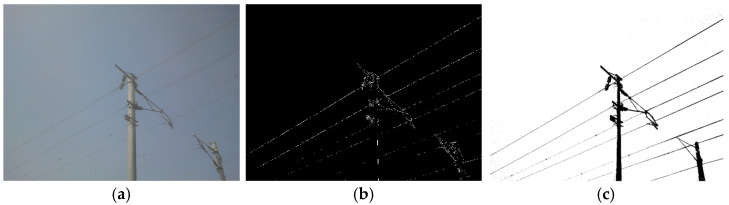
The experimental results of Scenario 3. (**a**) Original image. (**b**) Improved Ratio operator processing. (**c**) Four-direction chain code tracking processing.

To verify the effectiveness of the proposed algorithm, extraction experiments were conducted on the additional conductor targets in various background environments. The results are shown in Figure 15 and Figure 16. Figure 15a, Figure 16a, and Figure 17a display the original images. Figure 15b, Figure 16b, and Figure 17b show the corresponding edge extraction results using the improved Ratio algorithm. Figure 15c, Figure 16c, and Figure 17c illustrate the results after applying four-directional chain code tracking. These figures demonstrate the effectiveness of the improved Ratio algorithm in edge detection and the further enhancement provided by four-direction chain code tracking in complex backgrounds.

The images of the additional conductors in various scenes show that, after image preprocessing and enhancement, the edges of the conductor targets were effectively detected using the improved Ratio operator. Even in situations with low light intensity, complex backgrounds, and a high level of noise, the additional conductor targets remained clear and distinct. Following the four-direction chain code tracking, the continuity of the conductor targets was preserved, with the overall energy concentrated, yielding ideal results for extracting the conductor targets from the images.

### 4.3. Comparative Experiment

**Figure 18:**
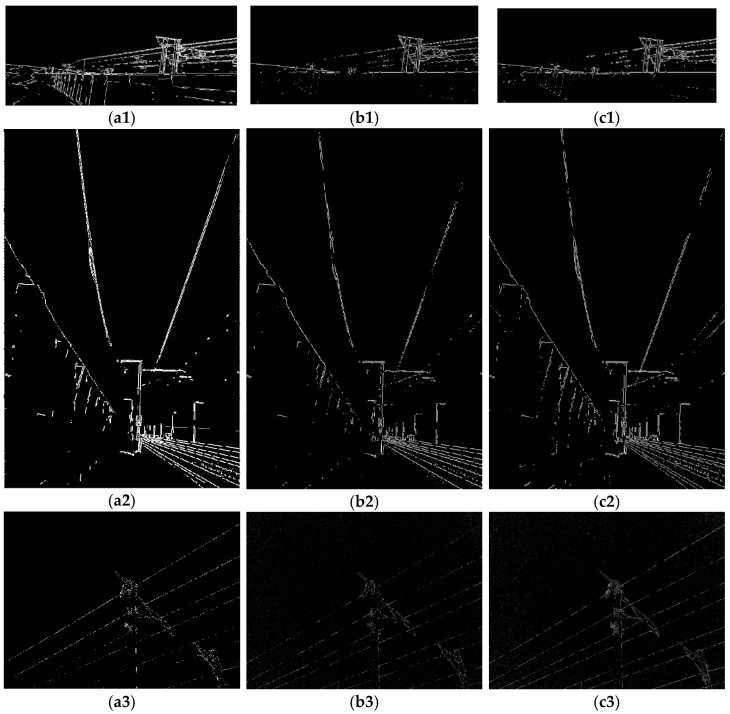
Comparative experiment. (**a1**) Ratio of Scenario 1; (**b1**) Roberts of Scenario 1; (**c1**) Sobel of Scenario 1; (**a2**) Ratio of Scenario 2; (**b2**) Roberts of Scenario 2; (**c2**) Sobel of Scenario 2; (**a3**) Ratio of Scenario 3; (**b3**) Roberts of Scenario 3; (**c3**) Sobel of Scenario 3.

To evaluate the effectiveness of the proposed improved Ratio operator in edge detection for the galloping monitoring of OCS additional conductors, we conducted comparative experiments with the Roberts and Sobel operators. The three methods were tested on video frames collected under diverse conditions, including low-light environments, high-noise scenarios, and varying background complexities. The experimental results indicate that in the scenario of additional conductor galloping detection, both the Roberts and Sobel operators exhibit significant issues of edge discontinuity and omission. Particularly in cases of complex backgrounds or insufficient lighting, these methods struggle to effectively extract the complete edge information of the conductors, rendering the detection results unsuitable for practical applications. In contrast, the proposed improved Ratio operator enhances pixel gradient ratios, maintaining a higher edge integrity and continuity in complex environments. This effectively addresses the limitations of existing methods in this scenario, making it more suitable for the task of monitoring additional conductor galloping, as shown in Figure 18.

At the same time, a detailed comparison was made with current popular end−to−end deep learning methods, such as YOLO and Faster R-CNN, and the discussion was expanded from the following aspects:

(1) End-to-end methods like YOLO and Faster R-CNN excel in target detection tasks but typically require substantial computational resources and high-performance hardware for optimal performance. In contrast, the proposed improved Ratio operator, with its lower computational complexity and hardware requirements, can run on standard industrial cameras and embedded devices. While it lacks the generalizability of end−to−end methods, it is particularly suitable for resource-constrained railway environments in strong wind zones. Experimental data show that YOLO models typically require more than 100 ms of inference time for high-resolution images, whereas the improved Ratio operator processes images within approximately 20 ms on the same hardware. This demonstrates the significant advantages of the Ratio operator in terms of real-time performance and hardware adaptability.
(2) The additional conductors have narrow pixel widths, occupy a small proportion of the image, and are situated in complex background noise, posing significant challenges to target detection algorithms. End-to-end deep learning methods are highly sensitive to large target detection but struggle with small target detection in the presence of background noise or lighting variations. The improved Ratio operator, by enhancing edge features through pixel gradient ratios, achieves higher accuracy and stronger robustness in detecting small targets, especially in low-light or noisy environments.
(3) Deep learning methods require extensively annotated datasets for training, which is time-consuming and costly in complex railway environments. The improved Ratio operator does not rely on large-scale training datasets and demonstrates strong adaptability, making it suitable for image processing tasks in various wind zones. Moreover, the working mechanism of the improved Ratio operator is more interpretable, as it clearly identifies the contributions of edge enhancement and noise suppression. In contrast, end-to-end methods often function as “black boxes”, making precise adjustments challenging.

In summary, the enhanced Ratio operator exhibits exceptional real-time performance, resilience, and adaptability in monitoring supplementary conductor galloping. It effectively meets the particular requirements of railway strong wind zone scenarios, rendering it an efficient and pragmatic monitoring solution.

## 5. Oscillation Monitoring Platform Testing and Analysis

By constructing an oscillation monitoring test platform, data on the oscillation of additional conductors are extracted from video monitoring, and the oscillation frequency and amplitude are analyzed to complete the monitoring and testing of the additional conductor oscillation state in the OCS.

### 5.1. Camera Calibration and Parameter Acquisition

To ensure that the monitoring of additional conductor oscillation along the Lanzhou–Xinjiang high-speed railway is fast, stable, reliable, and highly accurate, the calibration process is crucial. The currently established and widely used Zhang Zhengyou calibration algorithm is chosen to obtain the internal and external parameters and distortion coefficients of cameras [22].

**Figure 19:**
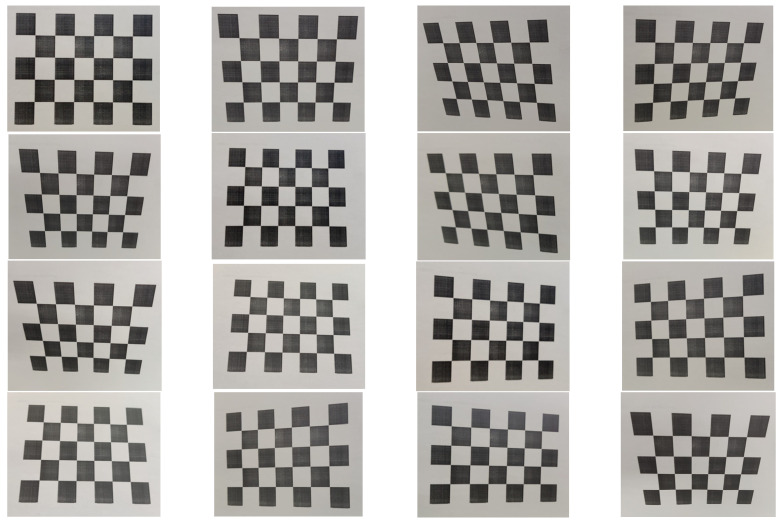
Checkerboard images shot from different angles.

**Figure 20:**
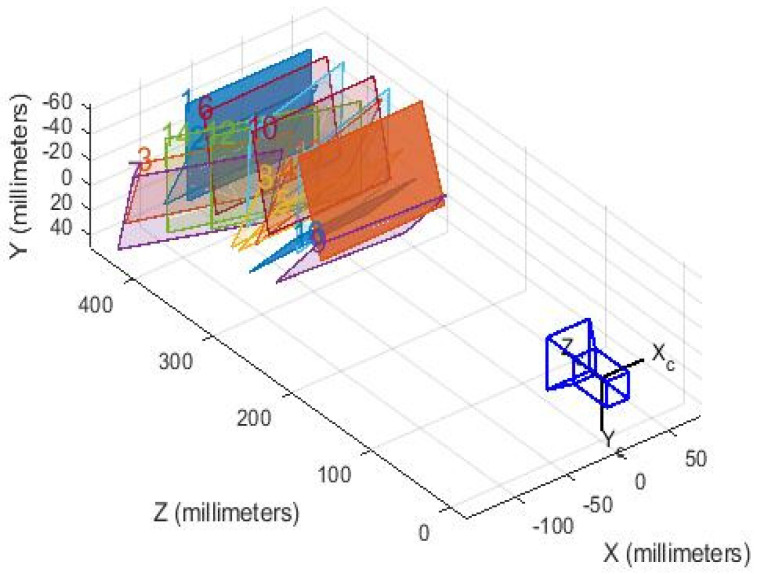
Calibration space stereogram.

In this experiment, a black-and-white checkerboard pattern with square sizes of 25 mm × 25 mm is used as the calibration target. Images of the checkerboard are taken from different angles, as shown in Figure 19. Sixteen images taken from different angles are used for parameter solving. The images are then imported into Matlab 2023b, and the Camera Calibrator program is used for calibration, as shown in Figure 20.

Based on the data from the oscillation monitoring test platform and the camera parameters, the camera calibration parameters were derived, yielding the internal parameters $M_{\mathrm{in}}$ and external parameters $M_{\mathrm{out}}$ as shown in Equations (1) and (2).

(1) $$M_{\mathrm{in}}=\left[\begin{array}{ccc} 2378.1 & 0 & 1956.1\\ 0 & 2044.5 & 1145.4\\ 0 & 0 & 1 \end{array}\right]$$

(2) $$M_{\mathrm{out}}=\left[\begin{array}{cccc} 1 & 0 & 0 & 0\\ 0 & 0.845 & -0.324 & -3501.3\\ 0 & 0.352 & 0.899 & 4002.46\\ 0 & 0 & 0 & 1 \end{array}\right]$$

### 5.2. Galloping Simulation Test for Additional Conductors

**Figure 21:**
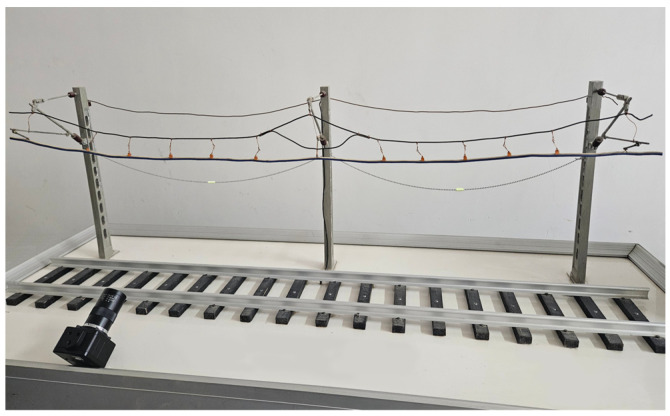
Video monitoring simulation test platform for OCS additional conductor oscillation.

In the laboratory environment, the conductor is fixed between two supports, as shown in Figure 21. A variable frequency drive controls the winch to induce conductor galloping, setting the same galloping frequency as the additional conductor of the Lanzhou–Xinjiang high-speed railway under actual conditions. The galloping state of the additional conductor is then monitored.

**Figure 22:**
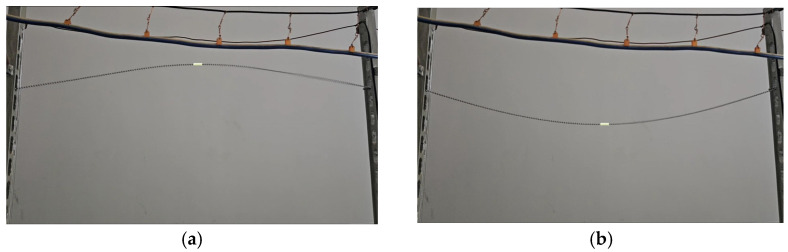
Frame of additional conductor galloping. (**a**) Highest point of additional conductor; (**b**) Lowest point of additional conductor.

Based on the coordinate relationship between the pixel coordinate system and the world coordinate system in the conductor galloping image, the spatial coordinates of the target feature points are obtained. To determine the feature point coordinates in the digital image and reduce the impact of camera distortion, camera calibration is performed using the Zhang Zhengyou calibration algorithm. The real-time transmitted galloping video images are processed frame by frame, and conductor target extraction is carried out, as shown in Figure 22a,b.

### 5.3. Galloping Data Analysis

When analyzing the galloping data of the additional conductors, two factors need to be considered: First, the data obtained from online monitoring must meet the objective requirements of the additional conductor galloping on the Lanzhou–Xinjiang high-speed railway. Second, the data should support the study of galloping mechanisms and anti-galloping technologies. For research on the galloping mechanisms and anti-galloping technologies of the additional conductors, the amplitude and frequency of galloping are the two most important and direct types of observational data. Therefore, in the galloping monitoring test platform, the amplitude and frequency of the conductor galloping are monitored.

To calculate the amplitude of galloping with the center position of the additional conductors as the target point, the following steps are performed:

(1) The world coordinate value of the target point when the conductor is static is calculated and used as the reference value.
(2) The pixel coordinates of the target point during conductor movement are obtained and converted into the world coordinate system position.
(3) The difference between the reference value and the coordinates during movement for each frame image is calculated to obtain the relative coordinates of the conductor movement.
(4) The obtained coordinates are transformed from the time domain to the frequency domain using Fourier transform.

**Figure 23:**
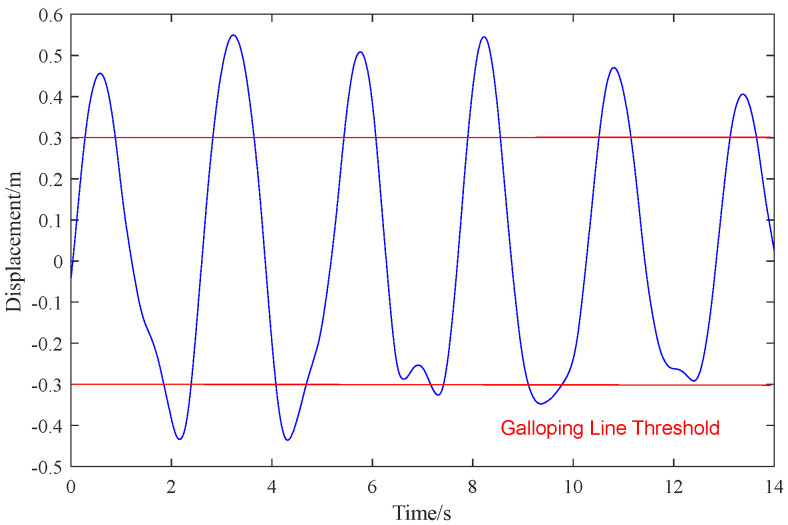
Time history curve of vertical displacement of target measuring point.

As shown in Figure 23, the vertical galloping trajectory of the target point within 15 s is consistent with the actual field situation. The displacement of the target point is stable and reliable, meeting real-time and stable monitoring requirements. The red line in the figure represents the set galloping limit value, which can be adjusted according to actual field conditions. When the galloping amplitude of the target point exceeds the set limit value, a signal is sent in time, and the information is transmitted to the monitoring center for early warning of the galloping state.

**Figure 24:**
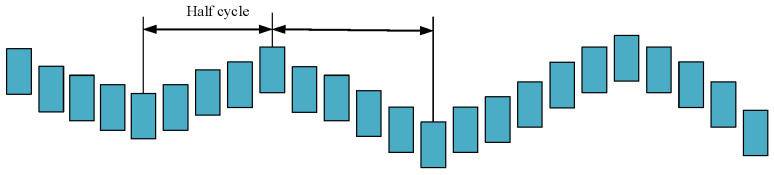
Schematic diagram of galloping frequency calculation.

In the galloping monitoring test platform, the video captured by the camera has a frame rate of 30 frames per second, with an image interval of 0.04 s per frame. The filming frequency is denoted as $f_{p}$. To determine the galloping frequency, the images with the lowest and highest points of the galloping motion are selected, then the nearest highest point (or lowest point) is found around these images. As shown in Figure 24, it is supposed that the number of images that find the interval between images is n, and the time interval between images is denoted as $1/f_{p}$. The time interval between the highest and lowest points of galloping is $n/f_{p}$. The galloping frequency can be calculated using Formula (3).

(3) $$f=\frac{1}{T}=\frac{1}{2n\times f_{p}}$$

**Figure 25:**
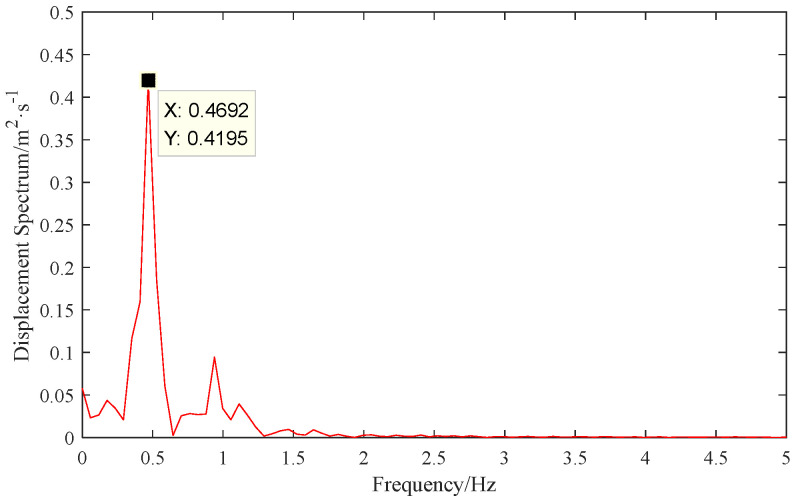
Spectrum curve.

The galloping frequency of OCS additional conductors remains consistent at different points on a single conductor. Therefore, extracting the galloping frequency for the additional conductors only requires calculating the frequency at one characteristic point. As shown in Figure 25, the galloping of the additional conductors primarily exhibits a first−order oscillation, and the fundamental frequency is 0.469 Hz, which matches the observed oscillation order in the field. Overall, by calculating the galloping amplitude and frequency of the additional conductors, the goal of extracting and analyzing galloping monitoring data is achieved. Additionally, the monitoring data obtained can be used to further complete the early warning and evaluation of the galloping state.

## 6. Conclusions

(1) Through the specific analysis of the OCS structure, the environment along the line, and the galloping characteristics of the additional conductor of the Lanzhou–Xinjiang high-speed railway, the layout of the video online monitoring system is designed, the appropriate data transmission and power supply modes are selected, and the camera layout of the monitoring system is completed.
(2) The pretreatment is used to enhance the image of conductor galloping, and the regional chain code clustering analysis is used to eliminate a lot of noise in the conductor edge detection, ensure the accurate and complete edge extraction of the additional conductor, and solve the problems of strand breaking, splitting, and edge overlap in the edge detection.
(3) The experimental results show that the online monitoring system for the galloping of the additional conductor can accurately extract and analyze the galloping displacement and frequency of the additional conductor, can efficiently grasp the galloping state of the additional conductor, and can be used in the patrol inspection of the OCS additional conductor of the Lanzhou–Xinjiang high-speed railway, with significant practical engineering application value.

## Figures and Tables

**Table 1:** Main performance parameters of batteries.

Battery Model	Battery Type	Voltage /V	Capacity /mAh	Discharge Temperature /°C	Diameter × Length /m^2^
LR6AA	Zinc–manganese alkaline battery	1.5	2500	−20/+60	14.5 × 50.5
D-4/3AA1300	Nickel–cadmium battery	1.2	1300	−20/+60	14.5 × 65.5
H-AA2100A	Nickel–metal hydride battery	1.2	2100	−10/+45	14.5 × 50.5
CR2450	Lithium battery	3.0	550	−20/+60	24.5 × 5.0
LC16340	Lithium-ion battery	3.7	880	−20/+60	16.5 × 33.7

## Data Availability

Data are contained within the article.
